# Malignant transformation in 5071 southern Taiwanese patients with potentially malignant oral mucosal disorders

**DOI:** 10.1186/1472-6831-14-99

**Published:** 2014-08-05

**Authors:** Yen-Yun Wang, Yen-Hsuan Tail, Wen-Chen Wang, Ching-Yi Chen, Yu-Hsun Kao, Yuk-Kwan Chen, Chung-Ho Chen

**Affiliations:** 1 Department of Clinical Research, Kaohsiung Medical University Hospital, Kaohsiung, Taiwan; 2 Instrument Technology Research Center, National Applied Research Laboratories, Hsinchu City 300, Taiwan; 3 Division of Oral and Maxillofacial Surgery, Department of Dentistry, Kaohsiung Medical University Hospital, Kaohsiung, Taiwan; 4 Division of Oral Pathology and Maxillofacial Radiology, Kaohsiung Medical University Hospital, Kaohsiung, Taiwan; 5 School of Dentistry, College of Dental Medicine, Kaohsiung Medical University, Kaohsiung, Taiwan; 6 Oral & Maxillofacial Imaging Center, Kaohsiung Medical University, Kaohsiung, Taiwan

**Keywords:** Oral potentially malignant disorders, Malignant transformation, Oral epithelial dysplasia, Oral squamous cell carcinoma

## Abstract

**Background:**

Oral cancers can be preceded by clinically evident oral potentially malignant disorders (OPMDs). The current study evaluated the rate and the time of malignant transformation in the various OPMDs in a cohort of patients from southern Taiwan. Parameters possibly indicative for malignant transformation of OPMDs, such as epidemiological and etiological factors, and clinical and histopathological features were also described.

**Methods:**

We followed-up 5071 patients with OPMDs—epithelial dysplasia with oral submucous fibrosis, epithelial dysplasia with hyperkeratosis/epithelial hyperplasia, hyperkeratosis/epithelial hyperplasia, oral submucous fibrosis, lichen planus, and verrucous hyperplasia—between 2001 and 2010 for malignant transformation.

**Results:**

Two hundred nineteen of these 5071 OPMD patients (202 men, 17 women; mean age: 51.25 years; range: 30–81 years) developed oral cancers (179 squamous cell carcinomas; 40 verrucous carcinomas) in the same sites as the initial lesions at least 6 months after their initial biopsies. The overall transformation rate was 4.32% (mean duration of transformation: 33.56 months; range: 6–67 months). Additionally, the mean time of malignant transformation was significantly shorter for lesions with than without epithelial dysplasia. The risk of malignant transformation was 1.89 times higher for epithelially dysplastic than non-dysplastic lesions. The anatomical site of OPMD and the presence of epithelial dysplasia were significantly associated with malignant transformation. The hazard rate ratio was 1.87 times larger for tongue lesions than for buccal lesions.

**Conclusion:**

Patients with OPMDs require long-term follow up.

## Background

Oral squamous-cell carcinoma (OSCC) accounts for more than 90% of oral malignancies and is the 11th most common cancer worldwide; it provides 3% of all newly diagnosed cancer cases [1,2]. Because of the high prevalence (16.5% in men) of betel quid chewers in Taiwan [3], OSCC is one of the leading types of cancer; it is the fourth most frequently occurring cancer and the fifth leading cause of cancer death in men in Taiwan [4]. The claims in recent research that the five-year survival rate of oral cancers is still low might be attributable to most cases of OSCCs being diagnosed at an advanced stage. The five-year survival rate of early stage (I and II) OSCC might be about 80%, but of advanced stage (III and IV) OSCC is only approximately 20% [5]. Because most cases of OSCC are preceded by clinically evident oral potentially malignant disorders (OPMDs), it is important to prevent malignant change for those patients diagnosed with OPMDs [6].

In our review of the English literature, we found only a few studies that focused on the malignant transformation potentials of various OPMDs. Hsue et al. [7] found that the malignant transformation rates of a cohort of 1458 patients with OPMDs were 5.4% for epithelial dysplasia with oral submucous fibrosis, 4.65% for epithelial dysplasia with hyperkeratosis/epithelial hyperplasia, 3.55% for hyperkeratosis/epithelial hyperplasia, 3.09% for verrucous hyperplasia, 2.1% for lichen planus, and 1.9% for oral submucous fibrosis; the overall malignant transformation rate was 3.02% and the average time for transformation was 42.64 months. In contrast, another study [8] reported that the malignant transformation rates of OPMDs in southern Taiwan were 24.24% for oral epithelial dysplasia, 20.00% for verrucous hyperplasia, 8.57% for hyperkeratosis/epithelial hyperplasia, and 0.00% for oral submucous fibrosis.

We investigated and updated the rate and the time of malignant transformation in a wide spectrum of potentially malignant oral epithelial lesions in a cohort of patients from Southern Taiwan. Moreover, parameters possibly indicative for malignant transformation of OPMDs, such as epidemiological and etiological factors, and clinical and histopathological features were described.

## Methods

The Oral Pathology Department and Oral & Maxillofacial (OMF) Surgery Department of the hospital not only provides services for nearly all the biopsied OMF lesions but is also the most heavily used referral center for patients with these lesions in southern Taiwan. A total of about 31,000 cases of OMF lesions from 2001–2010 was referred for histopathological examination and treated in the hospital. Therefore, despite the lack of national data of the various types of OMF lesions in Taiwan, the occurrence of such lesions among the cohort of referral patients treated in the hospital would be representative of in this geographical region.

In this follow-up study, we retrieved, from the hospital’s database, the medical records of patients diagnosed with epithelial hyperplasia/hyperkeratosis, verrucous hyperplasia, oral submucous fibrosis, and lichen planus between 2001 and 2010. In addition, oral epithelial dysplasia was established based on the following histopathological findings [9]: (1) basal layer hyperplasia; (2) nuclear enlargement and hyperchromastism; (3) loss of intercellular adhesion and normal polarization; (4) abnormal mitoses above the basal cell layer; (5) individual cell keratinization within the spinous layer; (6) cellular pleomorphism; (7) drop-shaped epithelial ridges; (8) irregular stratification; and (9) an altered nuclear-cytoplasmic ratio. Epithelial dysplastic lesions were subclassified as [a] mild (dysplastic changes within the lower one-third of the oral epithelium), [b] moderate (dysplastic changes within the lower two-thirds of the oral epithelium), and [c] severe (dysplastic changes greater than two-thirds but less than the entire thickness of the oral epithelium) [10]. All the pathological diagnoses were verified and validated by two board-certified oral pathologists.

This study complied with the Helsinki Declaration with the data collected after the approval of the Institutional Review Board of Kaohsiung Medical University Hospital (KMUH-IRB-2013-0300). In order to establish which of the selected patients with potentially malignant oral epithelial lesions would actually progress to develop oral cancer, periodical follow-up assessments were arranged for all patients with a frequency based on the clinical features and the need for therapy. Those lesions without dysplastic changes would be followed-up for every 6 months while those lesions with dysplastic alterations would be followed-up for every three months. Hence, in general, patients were followed-up at least once a year and all the patients within the data sets were followed until 30 June 2010. If a clinically malignant transformation was suspected, an additional biopsy was done to confirm the diagnosis of oral cancer. The duration required for malignant transformation is defined as the time from the initial biopsy of the OPMD to the additional biopsy that confirmed the diagnosis of oral cancer. Two criteria had to be met to confirm the actual malignant transformation to oral cancer: (1) a malignant transformation lesion had to occur at the same anatomical site as the precancerous lesion, and (2) a minimum of six months was required between the initial biopsy and the additional biopsy to confirm the malignant transformation.

JMP version 9.0.1 for Windows (SAS Institute, Cary, NC, USA) was used for all differences in the distribution of related factors in OPMDs and in the group with malignancies was estimated using a chi-square test. Time-to-event analysis involved estimating the probability that an event will occur at different points in time. The end point of follow-up in those developing cancer was the date of detection of oral malignancy, and in those lost to follow up were coded by the date of last visit, to arrive at "censored" data. Kaplan-Meier estimate was computed to estimate the probability of cancer-free survival. Cox proportional hazards model was applied to analyze the effect of single and multiple covariates in predicting cancer development. The results were considered significance when the p-value was < 0.05.

## Results

The data sets were verified in the cancer registry database of the hospital. The cancer registry committee in the hospital will check for those cases lost to follow up. Cases, which were lost to follow up, would be checked for whether there were malignant transformations with respect to medical charts or cancer registry database in the Ministry of Health and Welfare, The Executive Yuan, Taiwan at the end of the study. Consequently, in the current study, 5071 patients were diagnosed with various OPMDs (4299 males and 772 females; mean age for all: 48.87 years; mean age for males: 47.70 years; mean age for females: 55.33 years; range for all: 15–96 years; range for males: 18–96 years; range for females: 15–95 years). These 5071 patients with OPMDs had epithelial dysplasia with oral submucous fibrosis (n = 186, 3.67%), epithelial dysplasia with epithelial hyperplasia/hyperkeratosis (n = 957, 18.87%); oral submucous fibrosis (n = 994, 19.60%), lichen planus (n = 381, 7.51%); verrucous hyperplasia (n = 869, 17.14%), and hyperkeratosis/epithelial hyperplasia (n = 1684, 33.21%). Additionally, a majority of these OPMD lesions were in the buccal mucosa (60.51%), followed by the gingiva (13.65%), and the tongue (12.46%) (Table 1). Most of these 5071 patients presenting various OPMDs were males with the majority had the oral risk factors (alcohol drinking, betel-quid chewing, and cigarette smoking) (Table 2).

**Table 1:** Location of the 5071 potentially malignant oral mucosal disorders in the current study

**Histological diagnosis**	**Upper lip**	**Lower lip**	**Buccal**	**Mouth floor**	**Hard palate**	**Soft palate**	**Gingiva**	**Tongue**	**Total**
Epithelial dysplasia with oral submucous fibrosis	0 (0.00)	14 (7.53)	135 (72.58)	2 (1.61)	0 (0.00)	2 (1.61)	13 (6.99)	20 (10.75)	186 (100.00)
Epithelial dysplasia with hyperkeratosis or or epithelial hyperplasia	15 (1.57)	64 (6.69)	517 (54.02)	24 (2.51)	16 (1.67)	32 (3.34)	146 (15.26)	143 (14.94)	957 (100.00)
Oral submucous fibrosis	5 (0.50)	52 (5.23)	752 (75.65)	4 (0.40)	6(0.60)	10 (1.01)	111 (11.17)	54 (5.43)	994 (100.00)
Lichen planus	3 (0.79)	20 (5.25)	279 (73.22)	1 (0.26)	3 (0.79)	0 (0.00)	53 (13.91)	22 (5.77)	381 (100.00)
Verrucous hyperplasia	15 (1.72)	93 (10.70)	401 (46.14)	12 (1.38)	20 (2.30)	88 (10.13)	119 (13.69)	121 (13.92)	869 (100.00)
Hyperkeratosis or epithelial hyperplasia	12 (0.71)	88 (5.23)	966 (57.36)	22 (1.31)	25 (1.48)	49 (2.91)	250 (14.85)	272 (16.15)	1684 (100.00)
Total	50 (0.99)	331 (6.53)	3050 (60.15)	65 (1.28)	70 (1.38)	181 (3.57)	692 (13.65)	632 (12.46)	5071(100.00)

Data are N (%).

**Table 2:** Age, gender and oral risk factors of the 5071 potentially malignant oral mucosal disorders in the current study

		**Age**	**Gender**		**Alcohol drinking**		**Betel-quid chewing**		**Cigarette smoking**	
	**N**	**Mean ± Standard deviation**	**Male N (%)**	**Female N (%)**	**Yes N (%)**	**No N (%)**	**Yes N (%)**	**No N (%)**	**Yes N (%)**	**No N (%)**
Epithelial dysplasia with oral submucous fibrosis	186	47.74 ± 11.84	177 (95.16)	9(4.84)	141 (75.81)	45 (24.19)	166 (89.25)	20 (10.75)	164 (88.17)	22 (11.83)
Epithelial dysplasia with hyperkeratosis or epithelial hyperplasia	957	51.65 ± 12.69	828 (86.52)	129 (13.48)	702 (73.35)	255 (26.65)	852 (89.03)	105 (10.97)	824 (86.10)	133 (13.90)
Hyperkeratosis or epithelial hyperplasia	1684	48.70 ± 13.64	1422 (84.44)	262 (15.56)	1226 (72.80)	458 (27.20)	1489 (88.42)	195 (11.58)	1457 (86.52)	227 (13.48)
Oral submucous fibrosis	994	44.69 ± 12.43	914 (91.95)	80(8.05)	728 (73.24)	266 (26.76)	879 (88.43)	115 (11.57)	857 (86.2)	137 (13.8)
Lichen planus	381	51.08 ± 13.22	149 (39.11)	232 (60.89)	272 (71.65)	108 (28.35)	345 (90.55)	36 (9.45)	334 (87.66)	47 (12.34)
Verrucous hyperplasia	869	50.16 ± 12.09	809 (93.10)	60 (6.90)	645 (74.22)	224 (25.78)	747 (85.96)	122 (14.04)	735 (84.58)	134 (15.42)

Data are N (%).

Two hundred nineteen patients (202 males, 17 females) underwent malignant transformation to oral cancers (179 OSCCs; 40 verrucous carcinomas [VCAs]). The overall transformation rate was 4.32% and the mean duration of transformation was 33.56 months (Table 3). Nine (4.84%) of the 186 patients with epithelial dysplasia and oral submucous fibrosis in our cohort progressed to oral cancers (8 OSCCs; 1 VCA); 63 (6.58%) of the 957 patients with epithelial dysplasia and hyperkeratosis/epithelial hyperplasia also developed oral cancers (56 OSCCs; 7 VCAs). Moreover, a majority of these 72 patients with a malignant transformation had mild epithelial dysplasia (n = 61), and the remaining 11 patients had moderate (n = 6) or severe epithelial dysplasia (n = 5). Sixty-one (6.43%) of the 949 patients with mild epithelial dysplasia, progressed to oral cancer, 6 (5.56%) of the 108 patients with moderate epithelial dysplasia, and 5 (5.81%) of the 86 patients with severe epithelial dysplasia progressed to oral cancer. It is worth noting that 49 (2.91%) of 1684 patients with histologically innocent epithelial hyperplasia/hyperkeratosis underwent malignant transformation to oral cancers (37 OSCCs; 12 VCAs). In contrast, for the 59 (6.79%) OPMDs that progressed to oral cancers in 869 patients with verrucous hyperplasia, 44 were OSCCs and 15 were VCAs. Thirty-seven (3.72%) of the 994 patients with oral submucous fibrosis developed oral cancers (32 OSCCs; 5 VCAs). However, only 2 (0.52%) of 381 patients with lichen planus developed oral cancer (2 OSCCs) (Table 3).

**Table 3:** Number, percentage and mean duration of the malignant transformation for the 219 potentially malignant oral epithelial lesions with different histological diagnoses

**Histological diagnosis**	**Malignant transformation**	**Mean duration of malignant transformation**
	**N (%)**	**(months)**
Epithelial dysplasia with oral submucous fibrosis	9/186 (4.84)	42.47
Epithelial dysplasia with hyperkeratosis or epithelial hyperplasia	63/957 (6.58)	27.86
Oral submucous fibrosis	37/994 (3.72)	37.42
Lichen planus	2/381 (0.52)	8.07
Verrucous hyperplasia	59/869 (6.79)	33.49
Hyperkeratosis or epithelial hyperplasia	49/1684 (2.91)	36.55
Overall	219/5071 (4.32)	33.56

The most common sites for these 219 malignant transformation cases were the buccal mucosa (53.43%), the tongue (17.81%), and the gingiva (13.24%). Of the 39 cases on the tongue, the predominant sites were the tongue border (n = 27), the tongue ventrum (n = 3), and the tongue dorsum (n = 9) (Table 4).

**Table 4:** Locations of the 219 potentially malignant oral epithelial lesions with malignant transformation

**Histological diagnosis**	**Upper lip**	**Lower lip**	**Buccal**	**Mouth floor**	**Hard palate**	**Soft palate**	**Gingiva**	**Tongue**^**a**^	**Total**
Epithelial dysplasia with oral submucous fibrosis	0 (0.00)	0 (0.00)	6 (66.67)	0 (0.00)	0 (0.00)	0 (0.00)	0 (0.00)	3 (0.33)	9 (100.00)
Epithelial dysplasia with hyperkeratosis or epithelial hyperplasia	2 (3.17)	8 (12.70)	27 (42.86)	4 (6.35)	1 (1.59)	4 (6.34)	5 (7.94)	12 (19.05)	63 (100.00)
Oral submucous fibrosis	0 (0.00)	0 (0.00)	25 (167.57)	0 (0.00)	0(0.00)	2 (5.41)	5 (13.51)	5 (13.51)	37 (100.00)
Lichen planus	0 (0.00)	0 (0.00)	0 (0.00)	0 (0.00)	0 (0.00)	0 (0.00)	0 (0.00)	2 (100.00)	2 (100.00)
Verrucous hyperplasia	0 (0.00)	5 (8.47)	32 (54.24)	0 (0.00)	1 (1.69)	4 (6.78)	9 (15.25)	8 (13.56)	59 (100.00)
Hyperkeratosis or epithelial hyperplasia	0 (0.00)	2 (4.08)	27 (55.10)	0 (0.00)	0 (0.00)	1 (2.04)	10 (20.41)	9 (18.37)	49 (100.00)
Total	2 (0.91)	15 (6.85)	117 (53.42)	4 (1.83)	2 (0.91)	11 (5.02)	29 (13.24)	39 (17.81)	219 (100.00)

Data are N (%); ^a^ Tongue border: 27; tongue ventrum: 3; tongue dorsum: 9.

**Figure 1:**
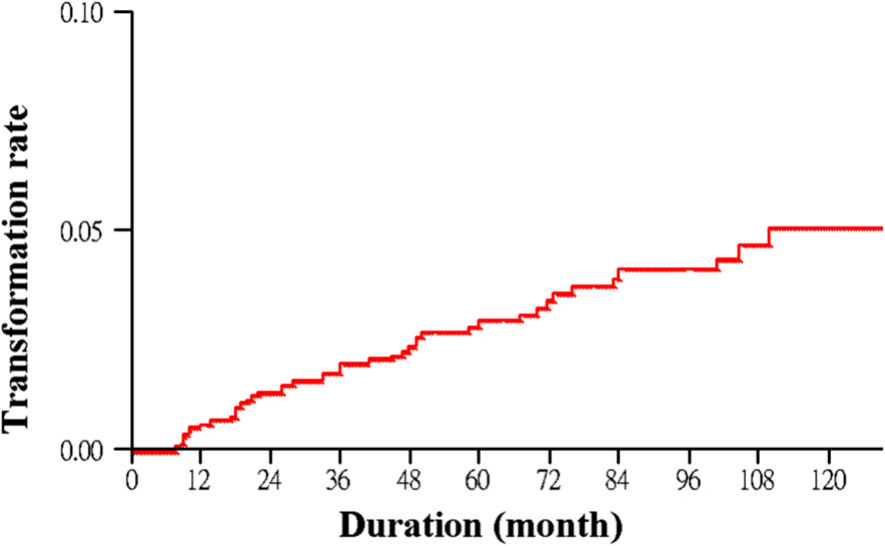
The annual malignant transformation rate of the current study.

**Figure 2:**
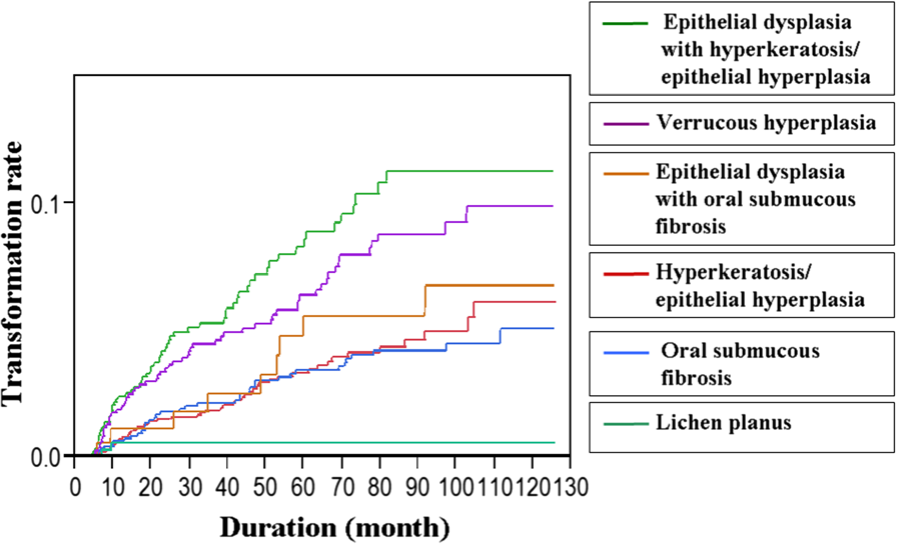
The annual malignant transformation rate of oral potentially malignant disorders (p < 0.0001; log-rank test).

Kaplan-Meier analysis estimated the 10-year transformation rate at 0.0689 (Figure 1; Table 5). The annual malignant transformation rates are shown in Figure 2. With the exception of the lichen planus lesions, the annual rates significantly increased over time (p < 0.0001).

**Table 5:** Annual malignant transformation rate (Kaplan-Meier estimates) of the current study

**Time (years)**	**Transformation rate**	**Standard error**	**95% confidence interval**
1	0.0119	0.0015	0.0093-0.0154
2	0.0232	0.0022	0.0192-0.0279
3	0.0290	0.0025	0.0244-0.0343
4	0.0385	0.0030	0.0329-0.0449
5	0.0461	0.0034	0.0398-0.0534
6	0.0543	0.0039	0.0471-0.0624
7	0.0594	0.0042	0.0517-0.0681
8	0.0615	0.0044	0.0535-0.0706
9	0.0669	0.0050	0.0578-0.0773
10	0.0689	0.0053	0.0592-0.0802

**Figure 3:**
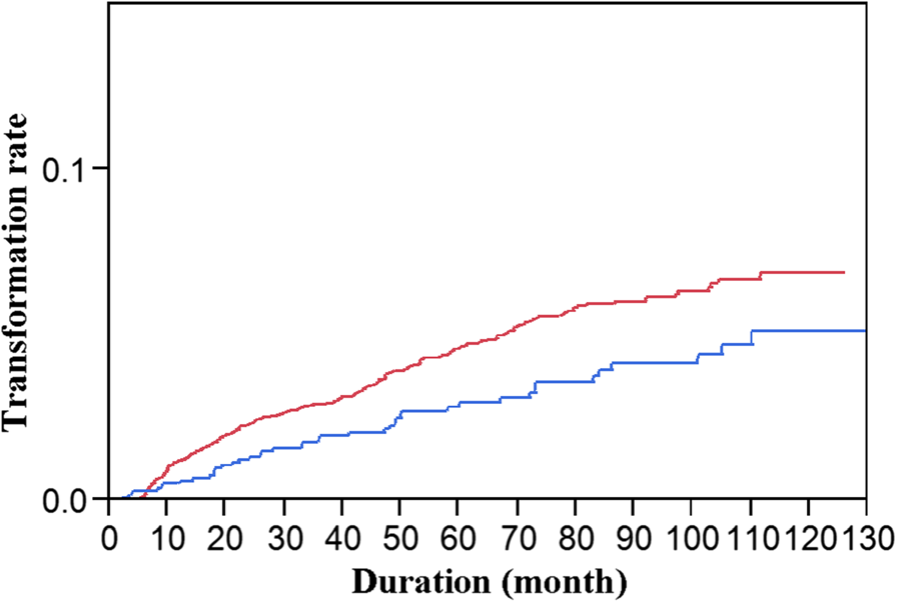
**Comparison of the annual malignant transformation rate of the current study with the previous study (1991–2001).** (Red line: The current study; blue line: Previous study) (p = 0.0104; log-rank test).

Further investigation with Cox proportional hazard regression analysis was performed (Table 6). In the multiple regression models, we considered the effect of age, gender, location, lesion type and oral risk factors on malignant transformation. We found that after adjusting for other factors, lesions located on the tongue were found to have a higher malignant risk, with a hazard ratio (HRR) of 1.87 (1.29-2.67) when compared to lesions located in the buccal mucosa. The malignant transformation risk for the group with epithelial dysplasia was 1.89 times more than those without epithelial dysplasia. But the degree of epithelial dysplasia was not statistically significant with malignant transformation. Moreover, the malignant transformation rate in the current study was significantly higher (p = 0.0104; log-rank test) (Figure 3) than that in our 1991–2000 study [7].

**Table 6:** Proportional hazards model of malignant transformation for related factors of the current study

**Factor**	**N (%)**	**Crude RR (95% CI)**	**p-value**	**ARR (95% CI)**	**p-value**
Location					
Buccal	3050 (60.15)	1		1	
Tongue	632 (12.46)	1.83 (1.26-2.60)	0.002	1.87 (1.29-2.67)	0.0014
Others	1389 (27.39)	1.30 (0.95-1.76)	0.09	1.33 (0.98-1.81)	0.07
Gender					
Female	772 (15.22)	1		1	
Male	4299 (84.78)	2.16 (1.36-3.68)	0.0007	2.15 (1.35-3.67)	0.0008
Epithelial dysplasia (ED)					
No	3928 (77.46)	1		1	
Yes	1143 (22.54)	1.98 (1.48-2.61)	< 0.0001	1.89 (1.39-2.54)	<.0001
Degree of ED					
Mild	949 (83.03)	1		1	
Moderate	108 (9.45)	1.96 (0.77-4.05)	0.14	1.18 (0.46-2.52)	0.70
Severe	86 (7.52)	2.03 (0.72-4.41)	0.16	1.08 (0.38-2.43)	0.88
Alcohol drinking					
No	1356 (26.74)	1		1	
Yes	3715 (73.26)	0.77 (0.58-1.02)	0.07	0.71 (0.53-0.96)	0.02
Betel-quid chewing					
No	593 (11.69)	1		1	
Yes	4478 (88.31)	0.83 (0.38-1.25)	0.38	1.71 (0.79-3.37)	0.16
Cigarette smoking					
No	700 (13.80)	1		1	
Yes	4371 (86.20)	0.73 (0.52-1.04)	0.08	0.42 (0.23-0.85)	0.02

CI, confidence interval; RR, risk ratio; ARR, adjusted risk ratio.

Finally, those patients older than 45 years at their first diagnosis showed significant higher malignant potential than the younger group of first diagnosis (p = 0.03); the male patients were significantly associated with malignant transformation when compared with female (p = 0.001); the oral risk factors were not associated with malignant transformation (Table 7).

**Table 7:** The analyses of age, sex and oral risk factors with malignant transformation in patients with OPMDs of the current study

	**Malignant transformation**		
	**Yes N (%)**	**No N (%)**	**p-value**
Age (years)			
≤45	71 (32.42)	1953 (40.25)	0.03
>45	148 (67.58)	2899 (59.75)	
Sex			
Male	202 (92.23)	4097 (84.44)	0.001
Female	17(7.77)	755 (15.56)	
Alcohol drinking			
No	70 (31.96)	1286 (26.50)	0.09
Yes	149 (68.04)	3566 (73.50)	
Betel-quid chewing			
No	30 (13.70)	563 (11.60)	0.33
Yes	189 (86.30)	4289 (88.40)	
Cigarette smoking			
No	40 (18.26)	660 (13.60)	0.057
Yes	179 (81.74)	4192 (86.40)	

## Discussion

Two important parameters should be considered when evaluating the potential for malignant transformation of OPMDs. First, the initial OPMD lesions should be confirmed using histopathological diagnoses; second, the amount of time it takes for the lesion to be transformed into a malignancy at the same location as the original OPMD lesion. In the present study, we included a wide spectrum of histopathologically diagnosed OPMDs and specified that the time from the initial presentation of the precancerous lesion to the malignant transformation must be at least six months and that the transformation must occur at the same site as the initial biopsy. We found that the overall malignant transformation rate of the OPMDs in our cohort was approximately 4%, which was lower than that of other studies [11-14] on the malignant transformation of precancerous lesions of leukoplakia. The reason for this difference may be that our study was based on the histopathological diagnoses of different kinds of precancerous oral lesions.

Epithelial dysplasia is a critical factor in determining malignant potential. In this study, 6.30% cases of epithelial dysplasia (72/1143: 83.03% (60) mild; 16.97% (12) moderate and severe) transformed into oral cancers, fewer than in another study [15] based on a relatively small Chinese sample in which the rate of malignant transformation was 26.8% (37/138: 28.6 (10/35) cases of moderate-to-severe epithelial dysplasia. One explanation for this difference might be that the majority of our cohort had mild epithelial dysplastic lesions, which usually have a lower risk of malignant transformation potential [15-17], and another might be that our sample of epithelial dysplastic lesions was 8.28 times larger (1143 vs. 138).

Using Cox proportional hazards regression analysis, we showed the potential effect of the degree of epithelial dysplasia on the malignant transformation rate and found that the risk of malignant transformation was 1.89 times higher for patients with dysplastic changes of precancerous lesions than for those without dysplastic changes. This is consistent with Amagasa et al. [12], who found that the rate of malignant transformation in patients with leukoplakia and epithelial dysplasia was much higher than that of patients without epithelial dysplasia (13.3% vs. 3.0%). Our finding was also compatible with a report from Northern Ireland [18], which indicated that 15% of their sample with epithelial dysplasia (25/167) had a malignant transformation, while only 1% without dysplastic changes (12/118) did, and that the risk of a malignant transformation for patients with severe epithelial dysplasia was significantly higher than for patients with mild epithelial dysplasia. Cowan et al. [18] found no significant difference in the risk between patients with moderate and mild epithelial dysplasia. These findings contrast with a report [15] on a Chinese population in which the malignant transformation risk for cases of moderate and severe epithelial dysplasia was 2.78 times higher than for cases of mild epithelial dysplasia (p = 0.002). This disparity might be due to the relatively low number of cases of moderate and severe epithelial dysplasia in our cohort because of the usual early local excision for the diagnosed cases of moderate and severe epithelial dysplasia in the hospital.

In the current study, 49 (2.91%) cases of histologically innocent hyperkeratosis/epithelial hyperplasia, with an average duration of 36.55 months, transformed into oral cancer. These data were compatible with two studies [7,8] on hyperkeratosis/epithelial hyperplasia cases with malignant transformation rates of 3.55% and 8.57% (mean duration: 41.30 months and 32.94 months), respectively.

Oral submucous fibrosis is a chronic condition of the oral mucosa in Asians, especially Indians [19,20]. In our cohort, 37 (3.72%) of 994 cases of oral submucous fibrosis transformed into malignancies with an average duration of 37.42 months, which was a higher transformation rate (1.9%) and a shorter mean duration for transformation (52.3 months) than in our 1991–2001 study [7]. This reflects not only the higher incidence rate of oral cancer within recent years, but also that the general public is more aware of Taiwan’s oral cancer pre-screening health promotion program and periodic clinical follow-up: For those persons (≥30 years old) who are the current/ex alcohol drinkers/betel-quid chewers/cigarette smokers are encouraged to receive oral examination for the possible presentation of OPMDs by the medical doctors (chiefly, Family Medicine, and Ear Nose and Throat specialties) as well as the dentists who have been trained for oral screening of the OPMDs from the local clinics or hospitals. The patients with suspected lesions of OPMDs will then be referred to the hospitals having oral biopsy service for histological confirmation. Furthermore, two months later, the government will check whether the referral patients have actually received oral biopsy procedures. Cases of malignant transformation also increased and the mean transformation time decreased. In contrast, of 66 patients followed-up for 17 years, 5 (7.6%) patients had OPMDs transform into OSCC in an Indian study [19], which was higher than in our study; this variation may reflect a larger sample size along with more stringent inclusion criteria in our study.

Oral lichen planus is an inflammatory mucocutaneous condition; its etiological factors are not yet completely certain [21]. It tends to occur in the buccal mucosa of 40-50-year-old women (female:male ratio = 2:1), [22]. In our study, women (average age at onset: 51.08 years) accounted for 60.89% of the cases of oral lichen planus, and 73.22% were in the buccal mucosa. The World Health Organization (WHO) recognizes oral lichen planus as an OPMD [6]. The transformation rate of oral lichen planus in the current study was 0.52%, which was lower than the 2.10% rate in our 1991–2001 study [7] but compatible with two other studies (0.4%, 0.5%, respectively) [23,24]. In the present study, the mean time needed for oral lichen planus to malignantly transform was 8.07 months, much shorter than for the other five types of OPMDs. This might be attributable to the small sample size (n = 2); in addition, one of these patients also had epithelial dysplasia.

Verrucous hyperplasia in the oral cavity has also been regarded as an OPMD and can develop into an OSCC or a VCA [25]. In the current study, 59 (6.79%) of 869 patients with verrucous hyperplasia developed oral cancer. The mean duration of 33.49 months was shorter than the 54.6 months in our 1991–2001 study [7], but the rate of malignant transformation was higher (10/324, 3.09%). In contrast, the rate of malignant transformation of our cohort was lower and the mean duration of transformation was shorter than in two other studies with relatively small sample sizes: Wang et al. [26] (10/60, 3.09%; 22.0 months) and Ho et al. [8] (9/44, 20.00%; 41.87 months).

We found that the average duration of malignant transformation was 33.6 months, shorter than in other studies [7,8]. Most of the OPMDs in our cohort were in the buccal mucosa (60.15%), the gingiva (13.65%), and the tongue (12.46%). Most of the 219 OPMDs with a malignant transformation in our cohort were in the buccal mucosa (53.43%), the tongue (17.81%), and gingiva (13.24%). However, we found that OPMDs in the tongue had a higher malignant transformation potential than those in the buccal mucosa (HRR = 1.83), which agreed with Amagasa et al. [12] and two studies from India [27,28].

We also found that the risk of malignant transformation was significantly higher for males than for females (HRR = 2.16). With the exception of two patients with lichen planus (one male, one female), all other types of malignant transformation showed that the male patients had a higher risk than did the female patients, which was consistent with the previous studies in India [27,28]. This might be because more males than females in Taiwan chew betel-quid, and more males habitually chew tobacco and smoke in India.

The three highest annual malignant transformation rates in the current study, in descending order, were epithelial dysplasia with hyperkeratosis/epithelial hyperplasia, verrucous hyperplasia, and epithelial dysplasia with oral submucous fibrosis. This indicates the significance of the malignant transformation of these three OPMDs and the potential impact of dysplastic changes on transformation. The annual malignant transformation rates of hyperkeratosis/epithelial hyperplasia and oral submucous fibrosis lesions were similar for the first 7 years. However, the transformation rate of hyperkeratosis/oral submucous fibrosis was significantly higher than that of oral submucous fibrosis after the 7th year, which indicated that despite the histological innocence of the hyperkeratosis/epithelial hyperplasia lesions, a long-term follow-up should be implemented.

Finally, the overall transformation rate of the current study (2001–2010) was compatible with that of our 1991–2001 study [7] (4.32% vs. 3.02%); however, a log-rank test showed that the annual transformation rate was significantly higher in the first 10 years in the current study than in our previous study.

## Conclusion

In the current study, we analyzed and updated the data of malignant transformation of various OPMDs in a cohort of patients from southern Taiwan. Moreover, our data indicated that patients with OPMDs need a long-term clinical follow-up and histopathological examination is an important predictor of cancer development to monitor the possibility of malignant transformation. Nevertheless, it should be cautioned that the current research was a follow-up study based in a Taiwanese hospital; a national based research is encouraged.

## Abbreviations

OPMD: Oral potentially malignant disorders; OMF: Oral and Maxillofacial; HRR: Hazard rate ratio; VCA: Verrucous carcinoma; OSCC: Oral squamous cell carcinoma; WHO: World Health Organization.

## Competing interests

The authors declare that they have no competing interests.

## Authors’ contributions

YHT, YYW, and YKC are the primary writers of the manuscript and participated in the study implementing. YKC conceived of the study, and had made substantial contributions to conception and design, and revised the manuscript critically for important intellectual content. YHT, YYW, and YKC assisted in statistical analysis, interpretation of data and draft the statistical analysis of manuscript. CHC, YKC, WCW, CYC, and YKC are the principal investigators of clinical studies in this project. All authors read and approved the final manuscript.

## Pre-publication history

The pre-publication history for this paper can be accessed here:

http://www.biomedcentral.com/1472-6831/14/99/prepub
